# Preparation of platelet-rich plasma as a tissue adhesive for experimental transplantation in rabbits

**DOI:** 10.1186/1477-9560-4-18

**Published:** 2006-09-28

**Authors:** Federico Luengo Gimeno, Silvia Gatto, José Ferro, Juan Oscar Croxatto, Juan Eduardo Gallo

**Affiliations:** 1 Department of Ophthalmology, Facultad de Ciencias Biomédicas, Universidad Austral, Av. Perón 1500, Pilar, B1629AHJ, Argentina; 2 Transfusional Service, Hospital Universitario Austral, Universidad Austral, Av. Perón 1500, Pilar, B1629AHJ, Argentina; 3 Department of Ophthalmic Pathology, Fundación Oftalmológica Argentina "Jorge Malbran", Azcuénaga 1077, Pb B, Buenos Aires, 1115 (falta el numero entero), Argentina

## Abstract

**Purpose:**

Platelet-rich plasma (PRP) is an autologous substance with adhesive properties. We aimed at developing and testing the efficacy of a method for PRP preparation in rabbits.

**Materials and methods:**

An *in vitro *study was carried out to obtain PRP from forty rabbits and to analyze the number of platelets and type of substance needed to trigger platelet activation. To induce platelet activation, 5%, 10%, 25% and 50% CaCl solutions were used. Then, an *in vivo *study was performed in twelve rabbits to test PRP adhesiveness in lamellar corneal graft. A control group made up of six rabbits underwent corneal transplantation without using PRP.

**Results:**

5% CaCl was the most effective concentration in activating PRP, with a mean time of 19 minutes. An attached corneal flap was seen 3 months after surgery. A detached corneal button was seen in all controls.

**Conclusion:**

Our method was able to produce rabbit-derived PRP with suitable properties for soft tissue adhesion. These results could be useful for researchers of the growing fields of tissue repair and experimental transplantation.

## Background

The platelet-rich plasma (PRP) is an autologous product that concentrates a high number of platelets in a small volume of plasma [1]. This product mimics the last step of the coagulation cascade, leading to the formation of a fibrin clot, which consolidates and adheres to the application site in a short period of time. Evidencing hemostatic and healing properties, PRP is able to hold tissues or materials in a required configuration [2]. Its biocompatible and biodegradable properties prevent the PRP from inducing foreign body reactions, tissue necrosis, or extensive fibrosis [3]. Absorption of the fibrin clot is achieved during wound healing within weeks following application [4-6]. PRP has been used in humans in different kinds of transplant procedures such as dentistry [7-9], orthopedics [10], maxillofacial surgery [11], plastic surgery [12,13] and ophthalmology [14]. In addition, PRP may be considered a carrier for biological active agents [15,16] and a healing substance causing less post-surgical pain [17,18].

Although the preparation of PRP in humans is well known [19], its preparation in rabbits is more difficult due to the reduced volume of blood and the smaller size of this animal. A blood sample, large enough to prepare PRP but without being lethal to the animal, should be collected. Reports in the literature differ about the intensity and duration of blood centrifugation, the number of platelets present in the PRP, and the use of thrombin or other factors to activate PRP [1,3,20]. Investigations also disagree on the puncture site and the quantity of blood that should be drawn from rabbits [20-22]. Furthermore, published studies lack an *in vivo *test of PRP adhesiveness in rabbits, animal frequently used in experimental transplantation.

The aim of our study was to obtain an ideal PRP solution for tissue adhesiveness and to test the effectiveness of the compound on lamellar corneal grafts using an in-vivo rabbit eye model.

## Materials and methods

The investigation was conducted in two steps. First, an *in vitro *study was performed to determine the best method for obtaining PRP from rabbits. An *in vivo *study was carried out then to evaluate the adhesiveness of the obtained PRP to soft tissue.

Forty adult New Zealand white rabbits weighing 3,000 grams each were used. Eighteen were female and twenty-two were male. The rabbits were treated in accordance with the guidelines of the ARVO Statement for the Use of Animals in Ophthalmic and Vision Research [23].

### *In vitro *study

Seventy-three blood samples from twenty-eight rabbits were collected. An 8.7 ml intracardiac blood sample was drawn under strict aseptic conditions. All animals received general anesthesia using a combination of midazolam 1 mg/kg intramuscular (Roche, Basel, Switzerland) and ketamine 70 mg/kg intramuscular (Fada, Buenos Aires, Argentina). The blood was aspirated with a 21 G needle. A 10 ml syringe preloaded with 1.3 ml of Anticoagulant Citrate Dextrose (ACD) solution was used to avoid coagulation. One millimeter was set apart for cell counting. The procedure was performed by two investigators (JF and SG).

Each blood sample was centrifuged for 15 minutes at 72 g at 4°C resulting in the three following layers: the inferior layer composed of red cells, the intermediate layer composed of white cells and the superior layer made up of plasma. The 6 ml plasma layer was centrifuged for another 5 minutes at 1006 g in order to obtain a two-part plasma: the upper part consisting of 5.5 ml of poor-platelet plasma (PPP) and the lower part consisting of 0.5 ml of platelet-rich plasma (PRP). The PPP was first aspirated to avoid its mixing up with the PRP. The PRP was then gently aspirated with another pipette and placed in a sterile tube. The PRP was thus prepared for activation by calcium chloride (CaCl), which inhibits the blood-thinning effect of ACD. After activation, PRP turned into a gel-like solution with adhesive properties and ready for use.

The coagulation time of PRP after CaCl instillation was evaluated in 250 μl samples placed in Eppendorf tubes with different CaCl concentrations. Coagulation was determined by the visualization of the clot at 20°C and carried out always by the same person (SG). The first analysis was performed using 5%, 10%, 25% and 50% CaCl concentrations, which were instilled into two PRP samples each from two different animals. A comparison was then made between 5% and 10% CaCl solutions in nine PRP samples, each obtained from five animals. Finally, a test was performed using 5% CaCl solution in 47 PRP samples from twenty one animals (table 1). The samples were observed for two hours and classified as non-coagulated if coagulation had not occurred during this time.

**Table 1:** The making process of platelet-rich-plasma in rabbits in vitro

	**1st step**				**2nd step**		**3rd step**
**CaCl concentration**	5%	10%	25%	50%	5%	10%	5%
**Number of animals**	2				5		21
**Number of PRP samples**	2	2	2	2	9	9	47
**PRP coagulation**	yes	yes	no	no	yes	yes	yes
**Coagulation time (minutes)**							
**- mean**	23	27	no	no	17	26	19
**- range**	18–25	25–31	-	-	10–20	20–50	7–39
**- st. Deviation**	-	-	-	-	3.9	10.2	7.4

The platelet count was carried out in the blood drawn and in the PRP sample with a Neubauer camera using the Brecher and Cronkite direct manual method [24,25].

### *In vivo *study

PRP preparation was performed in twelve rabbits using the same technique as in the *in vitro *study, and was activated by 5% CaCl to attach the corneal button as part of the autologous non-perforating corneal transplantation. Topical 5% iodopovidone was preoperatively instilled into the eye and over the eyelids, followed by topical 0.3% ciprofloxacin (Alcon, Sao Pablo, Brazil). Deep anterior keratectomy (one third of the stroma), using a 6-mm-diameter trephine, was done on right eyes under a ZEISS S5-Pro operating microscope (Zeiss C, Overckochen, Germany). The corneal button was excised according to Malbran's peeling off technique [26]. The flap was then soaked in autologous PPP and kept at 4°C for about 3 minutes. In the meantime, PRP was activated, transformed into a gel and placed over the stromal surface. The corneal flap was finally replaced on the stroma. Corneal graft adherence was manually verified at the end of the procedure and 30 minutes later using a delicate forceps. A partial tarsorraphy using three 5-0 silk sutures was carried out closing two thirds of the palpebral fissure. Twenty-one percent oxygen was administered to all animals for an hour after awakening. Topical 0.3% ciprofloxacin (Alcon, San Pablo, Brazil) and 0.1% dexamethasone (Poen, Buenos Aires, Argentina) were applied 4 times daily up to 14 and 21 days, respectively. The sutures of the partial tarsorraphy were removed on day 2.

A control group made up of six rabbits underwent lamellar corneal graft. In this group, the corneal button was gently placed on the stroma without using PRP. A tarsorraphy was also performed and removed on day 2.

Follow-up examinations were daily performed during the first week and weekly thereafter, using an operating microscope, a slit-lamp and surgical loupes. Examinations were carried out with special attention to corneal adhesiveness and inflammatory reaction. These features were photographically documented using a Nikon Coolpix 5700 Digital Camera (Nikon, Tokyo, Japan) on day 2, 7, 30 and 90. Histopathological analyses of the specimens were done 2, 7, 30 and 90 days after surgery. Histological specimens were stained with hematoxylin and eosin, periodic acid-Shiff (PAS) and Masson trichrome, and examined with a Nikon Eclipse E800 microscope (Nikon, Tokyo, Japan).

## Results

### *In vitro *study

All animals survived the blood extraction. Blood samples were free of clot. PRP was prepared in approximately 40 minutes. The number of platelets in PRP increased with respect to the number of platelets in the blood sample. In the PRP, the mean platelet concentration was 807.564 platelets/mm3 (range 622,000 – 1,350,000, SD 211,490) from an original vein concentration of 320,133 platelets/mm3 (range 280,000 – 408,000, SD 42,323). The resulting Platelet Enrichment Factor [20] was 152%. PRP treated with 25 % and 50% CaCl concentrations never coagulated whereas PRP treated with the 10 % CaCl concentration coagulated at 26 minutes. The mean coagulation time for 47 samples treated with the 5% CaCl concentration was 19 minutes (range 7 – 39, SD 7.4) (table 1).

### *In vivo *study

**Figure 1:**
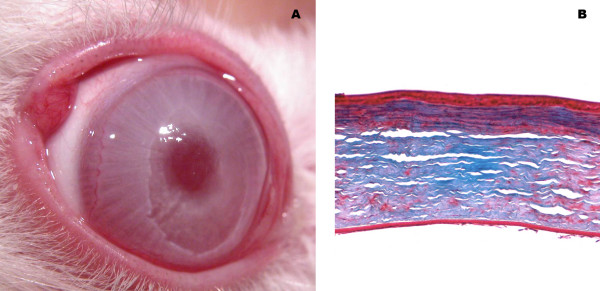
**Clinical and histopathological results 30 days after lamellar corneal transplantation**. An attached lamellar flap is seen with a suitable transparency at 30 days postoperative (A). This flap without epithelial defects is seen under Masson technique occupying the superficial third of the cornea.

**Figure 2:**
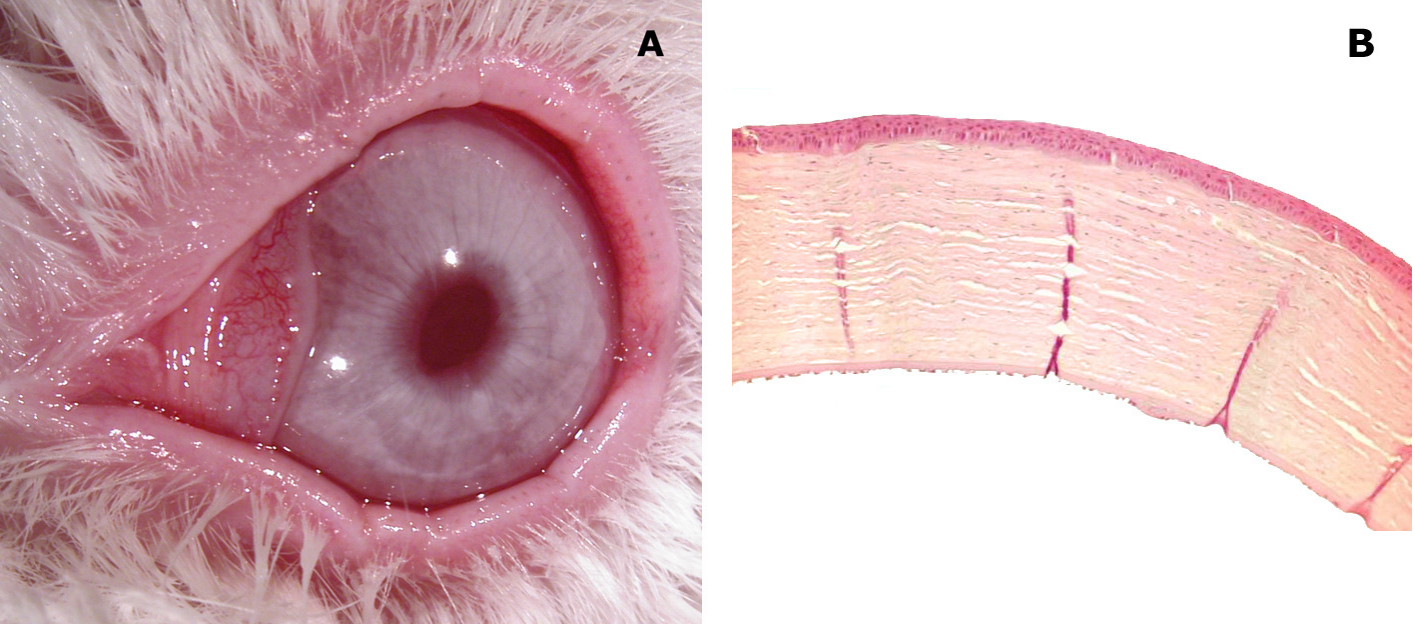
**Clinical and histopathological results 90 days after lamellar corneal transplantation**. Clear cornea (A) and attached lamellar flap with a normal appearence (B) (H&E stain, 60×) is seen at 90 days postoperative.

Animals with PRP showed corneal flap adherence within 20 minutes. An attached graft was seen in all rabbits and corneal clarity was observed from day 30 onwards (Figure 1). Clinical and histopathological examination of the eyes revealed no signs of inflammation. A normal interface between the graft and the host was found at 90 days post-operatively, as well as an intact epithelium (Figure 2). No flap complications such as diffuse lamellar keratitis, wrinkles, displacements or rotation of the flap were observed. A detached corneal flap was seen in all control animals after removing the tarsorraphy.

## Discussion

We developed a method for making PRP in rabbits, successfully used in non-perforating corneal transplantation. A comparison of methods used for preparing PRP is shown in Table 2. Blood coagulation problems are more frequently seen in female than in male rabbits [20]. This observation could influence rabbit PRP production. Anyhow, these differences do not seem to be relevant in humans [27]. It should be noted that the blood sample in our investigation had to be collected intracardiacally because we were not able to avoid blood coagulation by using different puncture sites, such as the ear or femoral vein. There is a wide range of variation in the intensity and duration of centrifugation among research studies. Ideal platelet counts differ among researchers. The machine and software used seem to play a role since rabbit derived platelets are smaller than those of humans [8]. Rabbits require specific software. That is why we carried out the platelet counts by direct observation. We thought that, by doing so, we would be able to get a more accurate count. Although some researchers have claimed that the best platelet concentration for producing PRP is 1 million/dl, this remains unclear. We tested the adhesiveness of PRP and can conclude that a Platelet Enrichment Factor of 152% seems to be useful at least for soft tissue adhesion.

**Table 2:** The comparison of methods used for PRP preparation in rabbits

**Study**	**Rabbit**			**Blood Samples**		**AC**	**Centrifugation**		**Hct**	**PRP**	**Platelets cells Count**		**EF**	**Activation type**	**Adhesive Test**
		
	**T**	**F**	**M**	**S**	**ml**	**Type**	**1st**	**2nd**	**%**	**ml**	**Vein**	**PRP**	**%**		
***Efeoglu et al [20]***	23	13	10	Vein	11	EDTA	300 g/10'	5000 g/5'	NS	NS	555.000	3.134.500	465	not done	not done

**Butterfield et al [22]**	12	0	12	Ear artery	21	citrate	150 g/20'	400 g/10'	NS	1	468.000	2.061.000	340	CaCl + BT	not done

**Hokugo et al [28]**	15	NS	NS	NS	10	citrate	2400 rpm/10'	3600 rpm/10'	NS	0,8	200.000	1.200.000	500	Fib + BT	not done

**Ohya et al [29]**	18	18	0	NS	30	NS	203 g/5'	1050 g/5'	NS	3	227.000	864.000	281	CaCl + BT	not done

**Jung et al [30]**	16	NS	NS	NS	9	citrate	150 g/20'	500 g/20'	NS	0,5	NS	NS	NS	CaCl + BT	not done

***Present study***	40	18	22	Heart	10	citrate	72 g/15'	1006 g/5'	41	1	320.133	807.564	152	CaCl	done

T: Total; F: Female; M: Male; Hct: Hematocrit; S: Source; AC: Anticoagulation; EDTA: Ethylenediaminetetraacetic acid; NS: Not Specified; CaCl: calcium chloride; Fib: Fibrinogen; BT: Bovine Thrombin; EF: Enrichment Factor

Observation: centrifugation values by Hokugo et al were reported in centrifuge rotor speed (rpm). Not enough data was available to make the conversion to times gravidity (g).

Other tissue adhesives have also been used in lamellar keratoplasty in rabbits and humans [28,29] and new synthetic glues like biodendrimer [30] and light-activated [31] adhesives are being tested in ophthalmology. Most of them have functional similarities but differ in origin and compounds. The major difference with PRP is that these commercial products are not autologous [1].

PRP is always autologous. Nevertheless, bovine thrombin has been used to activate PRP in previous studies without respecting the autologous aspect [22,32-34]. In our investigation, a chemical substance (5% CaCl concentration), was found suitable for triggering PRP activation. This could avoid the use of heterologous blood components which can cause immune reactions, and in turn, lead to negative results that could be falsely ascribed to PRP.

Several variables should be carefully monitored during PRP preparation. Special care must be placed on blood extraction tools, machines for blood centrifugation and CaCl solutions to prevent contamination of the compound. The process must be kept sterile and precisely suited to separate platelets from red blood cells. Unless platelet sequestration is done carefully and without causing any damage, platelets will no longer be able to secrete growth factors actively. PRP failure in previous studies may have resulted from non-adherence to these recommendations [21].

PRP, as tissue adhesive, has different applications in medicine [7]. It has also been used as carrier for pharmaceutical molecules, as antiangiogenic agents, and growth factors [32,35,36]. Gene therapy may benefit from PRP in future investigations since the presence of an adenoviral vector in the gel did not affect its properties [37]. It is also worth mentioning the recent use of PRP as cell supporter in corneal limbal epithelial cell transplantation [38].

In this experimental study in rabbits, PRP as an adhesive was able to attach the corneal button in the setting of non-perforating corneal transplantation. The corneal flap, about one third of the corneal thickness, remained attached up to three months post-operatively. Our survey on PRP preparation disclosed several difficulties that are yet to be overcome. This study could be taken as a guideline for rabbit-derived PRP preparation. We hope that the comments and data herein prove useful to researchers of the growing fields of platelet cell and experimental transplantation. Further studies are currently underway to evaluate the corneal wound healing response in the presence of PRP adhesive.
